# Effectiveness and safety of teclistamab for relapsed or refractory multiple myeloma: a systematic review and meta-analysis

**DOI:** 10.3389/fimmu.2025.1565407

**Published:** 2025-04-25

**Authors:** Wenze Li, Defeng Zhao, Yu Jiao, Weilin Dong, Ziyi Wang, Xiaojing Yan

**Affiliations:** ^1^ Department of Hematology, The First Affiliated Hospital of China Medical University, Shenyang, China; ^2^ Department of Thoracic Surgery, The First Affiliated Hospital of China Medical University, Shenyang, China

**Keywords:** teclistamab, relapsed or refractory multiple myeloma, meta-analysis, bispecific antibodies, systematic review

## Abstract

**Background:**

Multiple myeloma (MM) is a hematological malignancy with limited treatment options for patients with relapsed/refractory MM (RRMM). Teclistamab, a B-cell maturation antigen (BCMA) × CD3 bispecific antibody, has shown promising results in clinical trials and real-world studies.

**Methods:**

PubMed/MEDLINE, Web of Science, EMBASE, Cochrane Library, ClinicalTrials.gov, and meeting libraries were searched from inception to 14 November 2024. The assessed outcomes included overall survival (OS), progression-free survival, time to next treatment, duration of response, overall response rate (ORR), ≥complete response (≥CR), ≥very good partial response (≥VGPR), VGPR, partial response, and adverse events.

**Results:**

In total, 34 studies involving 4,064 patients were included. In pairwise meta-analysis, teclistamab demonstrated superior OS [hazard ratio (HR) = 0.69, 95% confidence interval (CI): 0.54–0.89; p = 0.037] compared to existing RRMM treatments. Real-world studies showed comparable ORR (62%, 95% CI: 58%–66%) but slightly lower survival outcomes, possibly because of shorter follow-up times and higher-risk populations. Subgroup analyses revealed enhanced efficacy with combination therapies (ORR: 85% vs 62%, p < 0.0001) and notable clinical benefits in the China cohort (≥VGPR: 77%, ≥CR: 58%). Safety profiles indicated manageable cytokine release syndrome and immune effector cell-associated neurotoxicity syndrome, though infection risks required vigilant management.

**Conclusions:**

Teclistamab continues to be a promising and effective treatment option for RRMM patients, including those previously exposed to BCMA-targeted therapies, and offers new hope for overcoming resistance and achieving better early disease control. Further research is needed to optimize its application in diverse populations, particularly in Asian cohorts.

**Systematic Review Registration:**

https://www.crd.york.ac.uk/prospero/#myprospero, identifier CRD42025633838.

## 1 Introduction

Multiple myeloma (MM) is a plasma cell malignancy characterized by uncontrolled overproduction of monoclonal immunoglobulin protein (M protein) and accounts for nearly 12% of hematological cancers (1, 2). Standard treatments for MM include proteasome inhibitors (PIs), immunomodulatory imide drugs (IMiDs), and anti-CD38 monoclonal antibodies. However, despite significant advancements in treatment options, MM remains an incurable disease. Available therapies for patients who are refractory to at least three drug classes (PIs, IMiDs, and anti-CD38 monoclonal antibodies) are limited, and their outcomes are generally poor (2–5). With a deepening understanding of disease biology, innovative therapeutic approaches continue to emerge.

In recent years, B-cell maturation antigen (BCMA)-directed therapies, including antibody-drug conjugates, chimeric antigen receptor (CAR) T-cells, and bispecific antibodies (BsAbs), have offered a new era of hope to patients with relapsed or refractory MM (RRMM). Teclistamab (JNJ-64007957, Janssen) is a bispecific antibody that targets the CD3 receptor complex on T cells and BCMA on MM cells (6). Preclinical studies have demonstrated the potent activity of teclistamab in MM cell lines, patient samples, and *in vivo* xenograft models (7). Teclistamab monotherapy was first demonstrated by the European Medicines Agency (EMA) on 23 August 2022 for the treatment of patients with RRMM who had received at least three prior lines of therapies including a PI, an IMiD, and an anti-CD38 antibody (8). Based on the positive response rates observed in the phase I/II MajesTEC-1 trial, the U.S. Food and Drug Administration (FDA) subsequently granted accelerated approval for teclistamab in patients with RRMM who had received at least four prior lines of therapy (9). Since the approval of teclistamab, many real-world studies have been conducted across various regions, including populations that did not meet the eligibility criteria of the MajesTEC-1 trial. Additionally, MajesTEC-1 also targeted another cohort of patients previously treated with BCMA-targeted therapies and reported promising efficacy (10).

With the increasing use of teclistamab in real-world settings, the number of related publications has also been steadily rising. Therefore, we conducted a comprehensive systematic review and meta-analysis aiming to compile and summarize the key data from all compared studies, clinical trials, and newly published real-world studies to deepen our clinical understanding of these therapies and provide significant insights into real-world physicians’ decision-making.

## 2 Materials and methods

This systematic review and meta-analysis adhered to the PRISMA guidelines. The analysis was registered in PROSPERO(CRD42025633838).

### 2.1 Data source and search strategy

Eligible studies were identified by searching databases including PubMed/MEDLINE, Web of Science, EMBASE, Cochrane Library, and ClinicalTrial.gov. The main international hematology meetings, including the American Society of Clinical Oncology (ASCO), the American Society of Hematology (ASH), and the European Hematology Association (EHA), were also searched to identify additional newly published relevant studies. The search only included articles published before 14 November 2024. Search terms included (“Multiple Myeloma” OR “Kahler Disease” OR “Plasma Cell Myeloma” OR “Myelomatose”) AND (“Teclistamab” OR “JNJ-64007957” OR “Bispecific antibody”). The specific search terms and strategies are listed in **Supplementary Table S1**.

### 2.2 Study selection

Potential trials were screened according to the following criteria: (1) patients diagnosed with RRMM; (2) randomized controlled trials (RCTs) and cohort studies; (3) teclistamab monotherapy or combined therapy was under investigation, with no restrictions on drug dosage; (4) clinical outcomes including any one or more of the following: overall survival (OS), progression-free survival (PFS), time to next treatment (TTNT), duration of response (DOR), overall response rate (ORR), ≥complete response (≥CR), ≥very good partial response (≥VGPR), VGPR, partial response (PR), and adverse events (AEs); (5) studies published in English language only.

The exclusion criteria were: (1) patients diagnosed with MM but not RRMM; (2) animal studies, comments, letters, reviews, and case reports; (3) the control arm was another CD3 × BCMA drug; (4) unpublished clinical trials; and (5) studies in which outcome data could not be extracted from texts, tables, or figures. Given the relatively short time since the approval of teclistamab, many clinical trials and real-world studies have presented their findings in the form of conference abstracts which were not excluded from the study.

### 2.3 Data extraction and quality assessment

Two authors (Li and Zhao) independently screened the literature and extracted the data, with any disagreements resolved by a third author (Jiao). The following extracted data were sorted into designed spreadsheets. (1) General study information including first author, publication years, article type, trial phase, National Clinical Trial (NCT) number, drug usage, and country. (2) Basic patients’ information included age, sex, refractory status, time to onset years, Eastern Cooperative Oncology Group (ECOG) scores, cytogenic risk status, International Staging System (ISS) stage, lines of previous therapies, and anti-BCMA exposure rate. (3) The main outcomes assessed were OS, PFS, TTNT, and DOR [hazard ratio (HR) and 95% confidence interval (CI)]; ORR, ≥CR, ≥VGPR, VGPR, and PR [relative risk (RR) or odds ratio (OR)]; and any-grade or grade ≥3 AEs, i.e., infection, neutropenia, anemia, cytokine release syndrome (CRS). and immune effector cell-associated neurotoxicity syndrome (ICANS). For articles that did not report OR or RR, the results were calculated using the MedCalc website (11). The extracted raw data can be found in **Supplementary Tables S2** and **S3**. To avoid duplicate data, only the most recent records were included and the long-term follow-up and subgroup analysis of the MajesTEC-1 trial were not included in the subsequent analysis. Most of the included studies were derived from conference abstracts, therefore, it was challenging and inaccurate to conduct a quality assessment.

### 2.4 Statistical analysis

Statistical analysis was performed using R 4.3.2 software. Because of the expected heterogeneity across the included studies, we chose a random-effects model over a fixed-effects model (12). HRs for survival outcomes (OS, PFS, TTNT, and DOR) and RRs and ORs for binary outcomes (ORR, ≥CR, ≥VGPR, VGPR, PR, and any-grade and severe-grade AEs) were calculated, along with their 95% CIs. The single-arm meta-analysis was conducted to calculate the overall rates of objective response and AEs of each treatment strategy from all eligible studies. Statistical heterogeneity among the studies was evaluated using the I^2^ statistic (13). Subgroup analyses were performed based on common characteristics across the included trials, such as region, study design, anti-BCMA exposure, and mono- or combined therapy. To address potential publication bias, weight functions were incorporated into the models to adjust the overall effect size estimates, and sensitivity analyses were conducted to assess their impact. Publication bias was corrected using a trim-and-fill method, which accounted for funnel plot asymmetry (14).

## 3 Results

### 3.1 Study selection and characteristics

**Figure 1:**
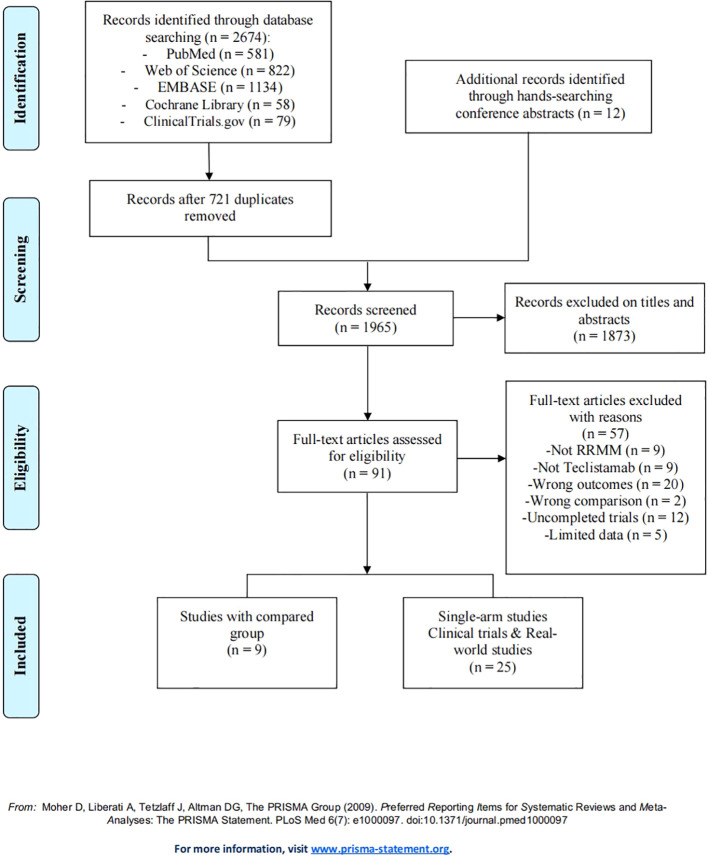
PRISMA flow diagram.

A total of 2,674 studies describing teclistamab for RRMM were found, with 581 studies from PubMed, 822 from Web of Science, 1,134 from EMBASE, 58 from Cochrane Library, and 79 from ClinicalTrials.gov. Furthermore, 12 additional records were identified through hand-searching conference abstracts. After removing 721 duplicate records, we reviewed the titles and abstracts of 1,065 articles, identifying 91 articles as potentially relevant for further analysis. After the application of the eligibility criteria to full-text review, 34 studies were included, with 9 studies that compared the efficacy and safety of teclistamab with currently and commonly used treatments for RRMM (15–23); 11 studies that were single-arm teclistamab clinical trials (9, 10, 24–32); and 14 studies that focused on real-world applications of teclistamab monotherapy (33–46). The complete screening process is listed in **Figure 1**, and the titles of excluded articles and the reasons for their omission are listed in **Supplementary Table S4**. There was a total of 4,064 patients in the included studies, with an average age of ~66 years. The baseline characteristics are summarized in **Table 1**.

Table 1Characteristics of the included studies.

**Table:** 

A)												
Comparative studies												
Author	Year	Article type	Drug name	Patient (n)	Age (years)	Sex (F/M, n)	Refractory status (5/4,3/other, n)	Time to onset (years)	ECOG scores (0/1, n)	Cytogenic risk status (high/standard/unknown, n)	ISS stage (I/II/III, n)	Lines of previous therapy
Bahlis, N. J (15).	2022	Conference abstract	Tec	37	NR	NR	NR	NR	NR	NR	NR	≥3 prior LOT
			Sel+ Dex	122								
Delforge,M (16).	2023	Conference abstract	Tec	165	NR	NR	NR	NR	NR	NR	NR	≥3 prior LOT
			RWPC	112								
Krishnan,A (17).	2023	Article	Tec	165	≥65: 47.9%	69/96	50/78/37	≥6: 50.9%	55/110	38/110/17	87/58/20	> 4 prior LOT: 52.7%
			RWPC	326	≥65: 48.1%	152/174	107/151/68	≥6: 52.3%	112/214	74/217/35	172/116/38	> 4 prior LOT: 56%
Mateos,M.V (18)	2023	Article	Tec	165	≥65: 47.9%	69/96	50/78/37	≥6: 50.9%	55/110	38/110/17	87/58/20	> 4 prior LOT: 52.7%
			Dara trials	264	≥65: 46.9%	133/131	75/120/69	≥6: 47.5%	119/145	58/179/27	142/94/28	> 4 prior LOT: 52.5%
Moreau, P (19).	2023	Conference abstract	Tec	165	NR	NR	NR	NR	NR	NR	NR	≥3 prior LOT
			BM	97
Moreau, P (20).	2023	Article	Tec	165	≥65: 47.9%	69/96	50/78/37	≥6: 50.9%	55/110	NR	87/58/20	> 4 prior LOT: 52.7%
			RWPC	302	≥65: 44.1%	132/170	108/129/64	≥6: 54.3%	93/209	NR	163/104/35	> 4 prior LOT: 55.9%
Rakesh Popat (22)	2024	Conference abstract	Tec	165	NR	NR	NR	NR	NR	NR	NR	≥3 prior LOT
			Pom+Dex	645
Dima, D (21).	2024	Conference abstract	Tec	45	Median: 68	19/26	Penta:26	NR	≥2:16	High:25	III:16	Median 6 prior LOT
			CAR-T	65	Median: 62	28/37	Penta:26	NR	≥2:9	High:19	III:17	Median 6 prior LOT
Song, J (23).	2024	Conference abstract	Tec	458	Median: 66	391/458	NR	NR	NR	NR	NR	≥4 prior LOT
			CAR-T	391

**Table:** 

B)																
Single-arm clinical trials																
Author	Year	Trial #	Study Design	Drug name	Usage	Patient (n)	Median age in years (range)	Median follow-up in months (range)	Sex (F/M, n)	Refractory status (5/4,3/other, n)	Time to onset (years)	ECOG scores (0/1, n)	Cytogenic risk status (n)	ISS stage (I/II/III, n)	Lines of previous therapy	Anti-BCMA exposed (%)
Moreau, P (9).	2022	MajesTEC-1: NCT03145181 NCT04557098	Open label, single-arm, phase 1-2 study	Tec	SC 1.5mg/kg	165	64.0 (33.0-84.0)	14.1 (0.3-24.4)	69/96	50/78/37	6 (0.8-22.7)	55/110	High: 38	85/57/20	Median 5 prior LOT	Not allowed
Rodríguez-Otero, P (24).	2022	NCT04108195	Phase 1b multicohort TRIMM-2 study	Tec + Dara	Dara: SC 1800 mg/schedule Tec: SC 1.5–3 mg/kg	46	67 (50–79)	7.2 (0.1-16.6)	24/22	NR	NR	NR	NR	NR	Median 6 prior LOT	15%
Cohen,Y. C (25).	2023	NCT04586426	Phase 1b RedirecTT-1 trial	Tec + Tal	NR	63	67 (39–81)	14.4 (0.5-21.9)	NR	Triple-class refractory: 49	NR	NR	High: 15	NR	Median 5 prior LOT	NR
Donk, N.1 (26)	2023	MajesTEC-1	Subgroup analysis	Tec	SC 1.5mg/kg	165	64.0 (33.0-84.0)	23	69/96	50/78/37	6 (0.8-22.7)	55/110	High: 38	85/57/20	Median 5 prior LOT	Not allowed
Donk, N.2 (26)	2023	MajesTEC-1	MajesTEC-1 Update	Tec	SC 1.5mg/kg	165	64.0 (33.0-84.0)	22	69/96	50/78/37	6 (0.8-22.7)	55/110	High: 38	85/57/20	Median 5 prior LOT	Not allowed
Searle, E (28).	2023	MajesTEC-2 NCT04722146	Open-label, multi-arm, phase 1b study	Tec+ Dara+ Len	TEC 0.72/1.5 mg/kg with step-up dosing+ Dara 1800 mg+ LEN 25 mg	32	62	5.78 (1.0-10.4)	4/28	NR	NR	NR	NR	NR	Median 2 prior LOT	Not allowed
Du juan (32)	2024	MajesTEC-1 China cohort	Open-label, single-arm, phase 1-2 study	Tec	SC 1.5mg/kg	26	66	15	19/7	Triple-class refractory: 16	NR	NR	High: 15	III:7	Median 5 prior LOT	Not allowed
Garfall, A. L (29).	2024	MajesTEC-1	MajesTEC-1Update	Tec	SC 1.5mg/kg	165	64.0 (33.0-84.0)	30.4	69/96	50/78/37	6 (0.8-22.7)	55/110	High: 38	85/57/20	Median 5 prior LOT	Not allowed
J Costa, L (30).	2024	MajesTEC-1	Subgroup analysis	Tec	SC 1.5mg/kg	165	64.0 (33.0-84.0)	30	69/96	50/78/37	6 (0.8-22.7)	55/110	High: 38	85/57/20	Median 5 prior LOT	Not allowed
Touzeau, C (10).	2024	MajesTEC-1 Cohort C	Open-label, single-arm, phase1-2 study	Tec	SC 1.5mg/kg	40	64 (32–82)	28 (0.7-31.1)	15/25	Triple-class refractory: 34	6.5 (1.1-24.1)	NR	High: 12	21/9/10	Median 6 prior LOT	100%
Donk, N (31).	2024	NCT05972135	Phase 2, multicenter, prospective OPTec study	Tec+Toci	SC 1.5mg/kg +IV Toci 8 mg/kg	24	72 (50-82)	8.1 (0.9-13.2)	NR	Triple-class refractory: 14	NR	NR	Standard:18	I/II: 23	Median 4 prior LOT	Not allowed

**Table:** 

**C)**													
Real world experiences													
Author	Year	Country	Article type	Patient (n)	Median age (range)	Sex (F/M, n)	Refractory status (n)	Time to onset (years)	ECOG scores (n)	Cytogenic risk status (%)	ISS stage (I/II/III, n)	Lines of previous therapy	Anti-BCMA exposed (%)
Uttervall, K. (33)	2021	Sweden	Conference abstract	17	62 (43-83)	7/10	Triple-class refractory: 15	NR	NR	NR	NR	Median 9 prior LOT	NR
Asoori, S (34).	2023	USA	Conference abstract	37	71 (50-89)	20/17	NR	NR	NR	NR	NR	NR	NR
Dima, D (35).	2023	USA	Conference abstract	102	75 (71-87)	NR	Triple-class refractory: 99	NR	NR	High: 58%	NR	Median 6 prior LOT	58%
Gordon, B (36).	2023	USA	Conference abstract	45	66 (45-88)	24/21	NR	4.9 (1.1-25.8)	0: 14 1: 19 ≥2: 12	High: 42.2%	11/13/12	Median 6 prior LOT	42.2%
Grajales-Cruz, A. F (37).	2023	USA	Conference abstract	22	66 (48-81)	9/13	Penta-class refractory: 11	NR	≥2: 3	High: 50%	III: 10	Median 8 prior LOT	100%
Maringanti, S. A (38).	2023	US, Greece, Spain	Conference abstract	80	69 (38–91)	36/44	Triple-class refractory: 49	NR	NR	High: 25%	NR	Median 6 prior LOT	50%
Dima, D (39).	2024	USA	Article	106	66.5 (35-87)	57/49	Triple-class refractory: 97	5.4 (0.5-20)	0-1: 71 2-4: 35	High: 59%	NR	Median 6 prior LOT	53%
Firestone, R. S (40).	2024	USA	Article	52	70 (39-88)	NR	Penta-class refractory: 35	6.3 (0.7-29)	0: 8 ≥1: 44	High: 33%	NR	Median 7 prior LOT	52%
Ghamsari, F (41).	2024	USA	Conference abstract	18	67 (50-83)	NR	Triple-class refractory: 18	NR	NR	High: 72%	NR	Median 6.5 prior LOT	39%
Graf, K. C (42).	2024	USA	Article	25	66 (37-78)	12/13	Penta-class refractory: 12	NR	NR	High: 36%	NR	Median 5 prior LOT	44%
Kawasaki, Y (43).	2024	USA	Article	All:27	69	12/15	NR	NR	1: 13	NR	NR	Median 5 prior LOT	NR
				1, 3, 5 days:23	69	9/14			1: 12				
				1, 4, 7 days:4	64	3/1			1: 1				
Mohan, M. (44)	2024	USA	Article	110	68 (37–89)	54/56	Penta-class refractory: 84	NR	NR	High: 62%	NR	Median 6 prior LOT	35%
Riedhammer, C (45).	2024	Germany	Article	123	67 (35-87)	53/70	Penta-class refractory: 74	6.5 (0.5-18.7)	NR	High: 36.8%	25/35/31	Median 6 prior LOT	37.4%
Tan, C. R (46).	2024	USA	Conference abstract	77	70 (63-77)	35/42	NR	NR	NR	High: 42%	NR	NR	NR

Sel, selinexor; Dex, dexamethasone; Tec, teclistamab; RWPC, real-world physician’s choice; LOT, lines of therapy; Dara, daratumumab; Pom, pomalidomide; BM, belantamab mafodotin; CAR-T, chimeric antigen receptor T cell; LEN, lenalidomide; Tal, talquetamab; Toci, tocilizumab; SC, subcutaneous; IV, intravenous; NR, not reported.

### 3.2 Efficacy and safety of teclistamab in compared studies

**Figure 2:**
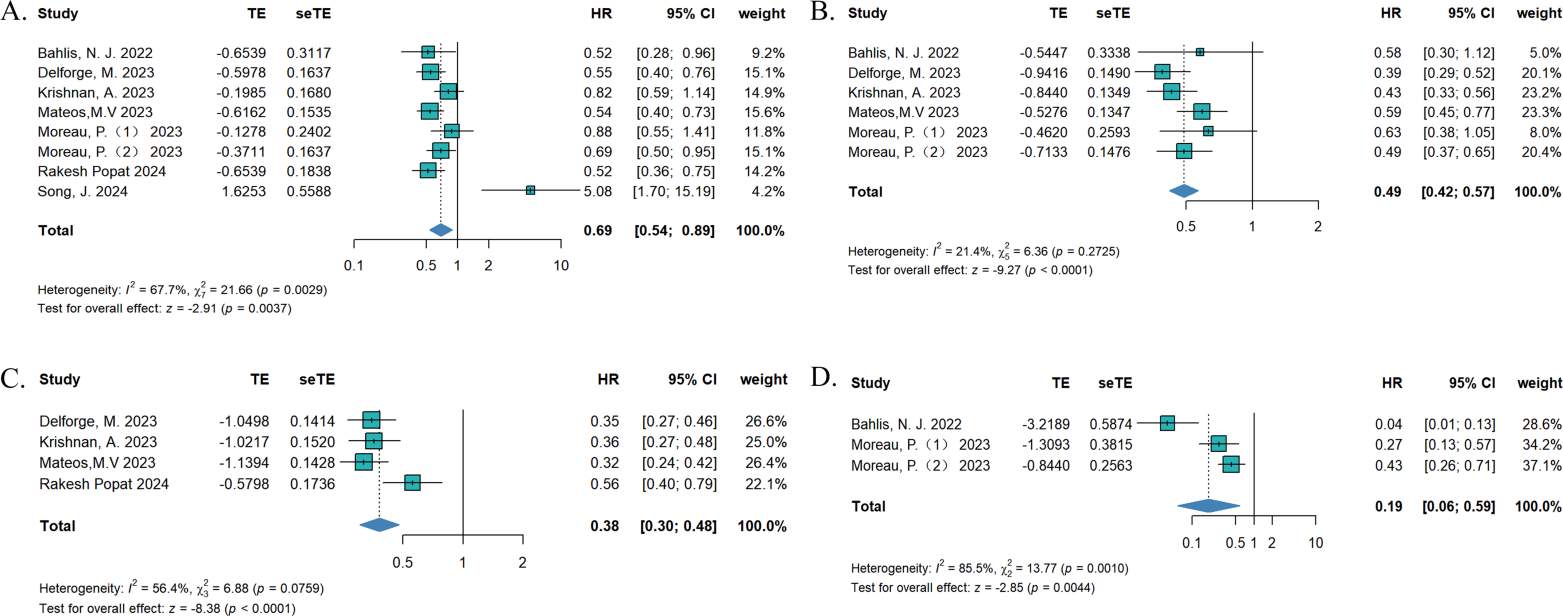
The pooled **(A)** OS, **(B)** PFS, **(C)** TTNT, and **(D)** DOR in patients treated with teclistamab in the compared studies.

**Figure 3:**
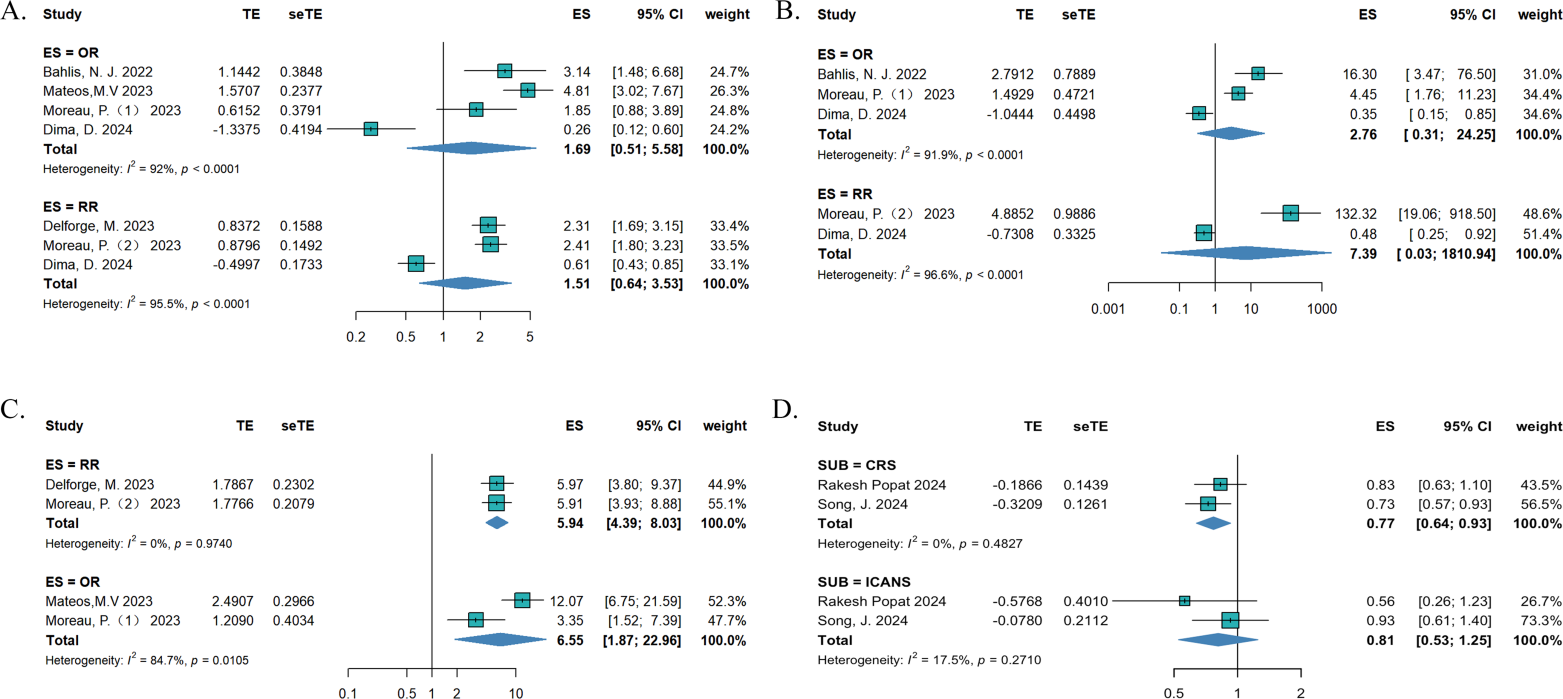
The pooled **(A)** ORR, **(B)** ≥CR, **(C)** ≥VGPR, and **(D)** AE in patients treated with teclistamab in the compared studies.

To compare the efficacy between teclistamab and currently used treatments for RRMM, we synthesized data on OS, PFS, TTNT, and DOR. The treatment measurements for the control group included selinexor plus dexamethasone (15), daratumumab (DARA) trials (18), belantamab mafodotin (19), pomalidomide plus dexamethasone (22), CAR-T (21, 23), and real-world clinical practice (16, 17, 20). Eight studies reported OS, six studies described PFS, four studies reported TTNT, and three studies reported DORs. In terms of survival outcomes, teclistamab demonstrated superior therapeutic advantages (**Figure 2**). The HR values for pooled OS, PFS, TTNT, and DOR were 0.69 [(95%CI: 0.54–0.89), p = 0.037], 0.49 [(95%CI: 0.42–0.57), p < 0.0001], 0.38 [(95%CI: 0.30–0.48), p < 0.0001], and 0.19 [(95%CI: 0.06–0.59), p = 0.0044], respectively. Considering the differences in variability in the data sources and the lack of baseline characteristic balancing in some studies, we conducted subgroup analyses of OS (**Supplementary Figure S1**). Four studies reported ORs (15, 18, 19, 21) and three studies describe RRs (16, 20, 21), respectively. As for the ORs, no significant differences were observed for ORR [effect size (ES) = 1.69, 95%CI: 0.51–5.58] and ≥CR (ES = 2.67, 95%CI: 0.31–24.25). As for RR, there was no statistically significant difference in ORR (ES = 1.51, 95%CI: 0.64–3.53) and ≥CR (ES = 7.39, 95%CI: 0.03–1810.94) as well (**Figures 3A, B**). However, after excluding the study that did not balance the baseline characteristics (21), regardless of whether OR or RR was reported, both ORR and ≥CR showed statistically significant differences (**Supplementary Figure S2**), suggesting that teclistamab achieved a higher response rate compared with existing treatment options. Compared to current treatments, teclistamab demonstrated superior outcomes in ≥VGPR (ES for RR = 5.94, 95% CI: 4.39–8.03; ES for OR = 6.55, 95% CI: 1.87–22.96) (**Figure 3C**). In the safety analysis, no significant differences were observed for any-grade ICANs (ES = 0.81, 95% CI: 0.53–1.25). However, compared to CAR-T, teclistamab was associated with lower incidences of any-grade CRS (ES = 0.77, 95% CI: 0.64–0.93) (**Figure 3D**).

### 3.3 Efficacy and safety of teclistamab in real-world events

**Figure 4:**
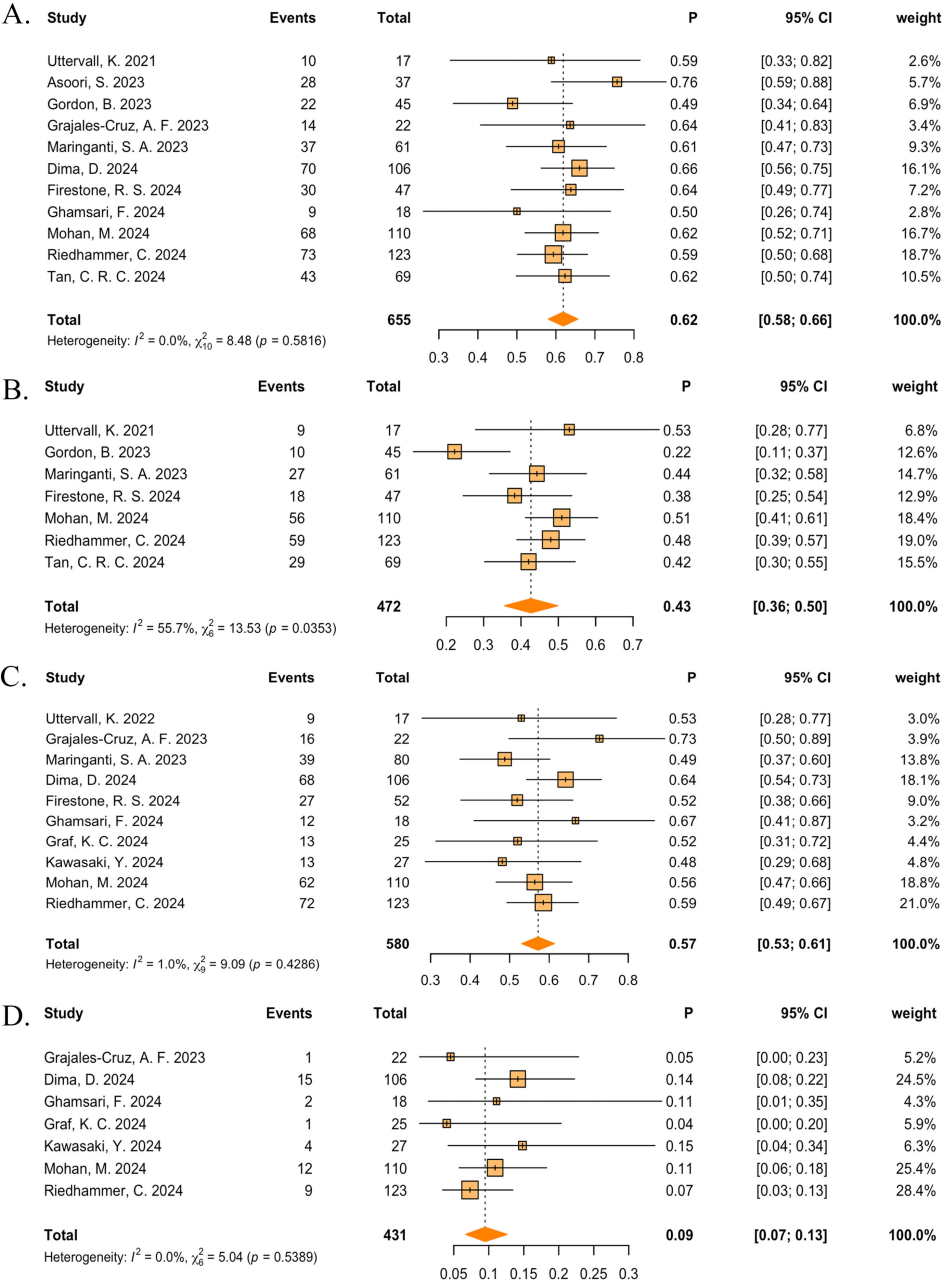
The pooled **(A)** ORR, **(B)** ≥VGPR, **(C)** any-grade CRS, and **(D)** any-grade ICANS in patients treated with teclistamab in the real-world studies.

In the real-world events meta-analysis, 11 studies reported ORRs (33–41), 7 studies reported ≥VGPRs (33, 36, 38, 40, 44–46), 6 studies reported ≥CRs (33, 37–39, 44, 45) and 7 studies reported PRs (33, 36, 38, 40, 44–46). Across all the teclistamab studies, regardless of region and ethnicities, the pooled ORR was 62% (95%CI: 58%–66%), ≥VGPR was 43% (95% CI: 36%–50%) (**Figures 4A, B**), ≥CR was 22% (95%CI: 16%–28%), and PR was 10% (95% CI: 7%–13%) (**Supplementary Figure S3**). The pooled incidence of any-grade CRS was 57% (95%CI: 53%–61%) and any-grade ICANs was 9% (95%CI: 7%–13%) (**Figures 4C, D**). Other AEs were all pooled and are shown in **Supplementary Figure S4**.

### 3.4 Subgroup analysis of single-arm studies

First, to explore whether there were differences in efficacy and safety between teclistamab monotherapy and combination therapy, we conducted a subgroup analysis. Three clinical trials reported a combination therapy with teclistamab (24, 25, 28). The combination therapy group displayed a higher ORR (85% vs 62%, p < 0.0001) and a higher ≥VGPR (68% vs 48%, p = 0.0247) than monotherapy while showing a similar ≥CR with monotherapy (29% vs 28%, p = 0.8481) (**Supplementary Figure S5**). For AEs, no statistically significant differences were observed for any-grade anemia, CRS, infection, and neutropenia, and grade ≥3 anemia, CRS, ICANS, infection, and neutropenia (**Supplementary Figure S6**).

Second, there were no significant differences in efficacy between clinical trials and real-world studies, except for ≥VGPR (60% vs 48%, p = 0.0247) and ≥CR (41% vs 22%, p = 0.0052). The pooled ORR, VGPR, and PR were 63% versus 62% (p = 0.7992), 19% versus 25% (p = 0.4222), and 13% versus 10% (p = 0.8171), respectively. The forest plot can be found in **Supplementary Figure S7**. For hematological AEs, compared to clinical trials, real-world studies exhibited a lower risk of neutropenia (any-grade: 79% vs 45%, p = 0.0017; grade ≥3: 66% vs 33%, p = 0.0001). No significant differences were observed in the risk of anemia (any-grade: 63% vs 66%, p = 0.8549; grade ≥3: 39% vs 23%, p = 0.1003). For non-hematological AEs, real-world studies had a lower risk of any-grade CRS (79% vs 58%, p = 0.0301), a lower risk of infection (any-grade: 81% vs 47%, p = 0.0002; grade ≥3: 50% vs 24%, p = 0.0003), and a higher risk of ICANS (any-grade: 3% vs 10%, p= 0.0297) (**Supplementary Figure S8**).

Third, compared with a Western population, the China cohort demonstrated superior ≥VGPR (77% vs 45%, p = 0.0021) and ≥CR (58% vs 25%, p = 0.0020). There were no statistically significant differences in ORR (77% vs 62%, p = 0.1098). As for AEs, the China cohort experienced a higher rate of any-grade anemia (88% vs 60%, p = 0.0078), any-grade CRS (96% vs 60%, p < 0.0001), any-grade infection (96% vs 53%, p < 0.0001), and any-grade neutropenia (96% vs 55%, p < 0.0001). The forest plots are listed in **Supplementary Figure S9**.

Fourth, five studies reported the outcomes of teclistamab treatment in populations previously exposed to BCMA-targeted therapies (10, 38–40, 45), and six studies reported the outcomes of populations with no prior BCMA exposure (9, 32, 38–40, 45). The non-BCMA-exposed group displayed a higher ORR than the anti-BCMA-exposed group (67% vs 56%, p = 0.0205). For the anti-BCMA exposed group, the pooled ≥VGPR, ≥CR, VGPR, and PR were 46% (95%CI: 38%–55%), 28% (95%CI:19%–38%), 25% (95%CI: 11%–43%), and 23% (95%CI: 2%–55%), respectively. For the non-BCMA exposed group, the pooled ≥VGPR, ≥CR, VGPR and PR were 59% (95%CI: 42%–75%), 41% (95%CI: 30%–52%), 19% (95%CI: 14%–25%) and 4% (95%CI: 0%–12%), respectively. The results are shown in **Supplementary Figure S10**. No statistical differences were observed for AEs (**Supplementary Figure S11**).

### 3.5 Sensitivity analysis and publication bias

The sensitivity analysis of OS in the compared studies confirmed that when Song, J. (2024) was individually excluded, I^2^ changed to 23.8% (**Supplementary Figure S12**). For PFS, when each trial was individually excluded, only minimal changes were observed. Egger’s test showed no indication of publication bias for OS (p = 0.0751) and PFS (p = 0.4676) (**Supplementary Figure S13**).

## 4 Discussion

MM is the second most common hematological malignancy, and during its course, almost all patients experience one or more relapses (47). Patients with RRMM frequently face the challenges of undergoing multiple lines of treatment with limited clinical success, underscoring the need to explore innovative and effective therapeutic options (48). Teclistamab, a BCMA × CD3-directed bispecific antibody, showed high response rates and durable remissions in the MajesTEC-1 trial in patients with RRMM. In this large-scale systematic review and meta-analysis, we quantified the reported efficacy and safety of teclistamab in RRMM.

In the pairwise meta-analysis, compared with existing treatment options for RRMM, teclistamab demonstrated superior efficacy, except for two articles comparing teclistamab with CAR-T therapy (21, 23). The inferior responses and survival outcomes of the teclistamab group may have been due to the variations in baseline characteristics across populations and can be explained by the more aggressive disease biology, as evidenced by poorer performance status, and higher rates of high-risk cytogenetics. Regarding AEs, CRS was only reported with CAR-T cell therapies. Despite the more aggressive disease biology observed in the teclistamab group, the incidence of CRS was still lower compared to the CAR-T group, suggesting that teclistamab offers better tolerability, even in patients in poorer physical condition. According to preliminary results from the KarMMa study, idecabtagene vicleucel (ide-cel) demonstrated an ORR of 73% in patients who had received at least three prior lines of therapy (8). In the CARTITUDE-1 trial, ciltacabtagene autoleucel (cilta-cel) showed an ORR of 98% in patients treated with at least three prior lines of therapy (49). Although CAR-T therapy has shown impressive response rates, the interval between leukapheresis and CAR-T cell infusion can pose challenges, especially for patients with rapidly progressing disease who may experience worsening cytopenia, progressive organ dysfunction, and declining functional status. In contrast, teclistamab offers the advantage of rapid treatment initiation in cases of rapidly progressing disease and demonstrates better tolerability in patients in a compromised physical condition (45). Therefore, given that both CAR-T and T-cell engagers (TCEs) have their respective advantages and disadvantages, and in the absence of direct head-to-head comparisons, it is recommended that CAR-T therapy be prioritized for eligible candidates when both CAR-T and TCE are equally accessible. However, TCEs, due to their greater accessibility and quicker initiation, should be preferred for patients with rapidly progressing disease who are unlikely to tolerate leukapheresis or bridging therapies. This recommendation is based on the activity data of TCEs following CAR-T treatment, and the longer treatment-free interval after CAR-T therapy, which provides more time for the administration of additional treatment options when relapse occurs (50).

In the real-world study analysis, the pooled ORR for the entire cohort was 62%, which was nearly equal to the ORR of 63% in the MajesTEC-1 trial (9). It is noteworthy that almost half of the real-world studies’ patients did not meet the key inclusion criteria of the clinical trial and also had high-risk features such as ISS 3, high-risk cytogenetic aberrations, extramedullary disease (EMD), or high bone marrow infiltration. This could explain why the median PFS in the real-world studies ranged from 5.4 to 12.7 months, with most results slightly lower than the 11.3 months observed in MajesTEC-1. Additionally, the lower rates of ≥VGPR (43%) and ≥CR (22%) observed in the real-world studies could also be attributed to these baseline differences, as patients with more high-risk features tend to have poorer responses. Other factors contributing to these differences could include the shorter median follow-up time in real-world settings, as responses have been shown to deepen over time, and differences in treatment adherence between real-world patients and those in clinical trials. Common AEs of BsAbs therapy included CRS, infections, and neutropenia. In the real-world studies, same as MajesTEC-1, CRS and ICANS were predominantly low-grade and effectively manageable in most cases. The pooled any-grade CRS rate was 57%, lower than that reported in the MajesTEC-1 trial (72%), and could be well managed by antipyretics, analgesics, corticosteroids, and tocilizumab. However, our results demonstrate that the risk of severe CRS and ICANS (grade ≥3) with teclistamab in the real-world setting is higher compared to that noted in clinical trials (1.9% vs 0.6%; 2% vs 0.6%). This is mainly because of the higher tumor burden, which is an important predictor of severe CRS with BsAbs and CAR-T therapy (51). Moreover, cytopenia in real-world studies, such as neutropenia and anemia, were mainly high-grade, which may lead to an increased risk of serious opportunistic infections. Though the any-grade infection rate was lower than MajesTEC-1 (47% vs 76.4%), this may have been associated with the shorter follow-up time in the real-world studies or the primary intravenous immunoglobulin (IVIG) prophylaxis administration (44, 52). Our analysis showed that grade ≥3 infections occurred in 24% of patients treated with teclistamab. The common infections were COVID-19, pneumonia, and upper respiratory tract infection. Dima and colleagues reported three deaths from severe infection while on teclistamab without any evidence of disease progression (39), hence, there is a need for close surveillance and adequate preventive measures for the high rates of infections (53). Better infection risk management is highly suggested for the future use of teclistamab to prevent patients from serious or even fatal outcomes.

This study also presented interesting findings in the subgroup analysis. First, compared to teclistamab monotherapy, the ORR rate increased from 63% to 78% when combined with DARA and further rose to 90% when combined with both DARA and lenalidomide (LEN). Both DARA and LEN possess immunomodulatory effects that may enhance the activity of teclistamab. This might be explained by the immunomodulatory effects of LEN when combined with DARA. The combination can enhance T and NK cell-mediated cytotoxicity and induce *in vivo* T cell proliferation (54). Furthermore, teclistamab can recruit CD3+ T cells to the vicinity of BCMA-positive clonal plasma cells, enhancing targeted cytotoxicity against myeloma cells (6). As for AEs, the combination therapy shows no statistic differences in any-grade anemia, any-grade CRS, any-grade infection, any-grade neutropenia, grade ≥3 anemia, grade ≥3 CRS, grade ≥3 ICANS, grade ≥3 infection, and grade ≥3 neutropenia, and an even lower rate of any-grade ICANS was observed in the pooled studies. These results indicate that the combination therapy had tolerable safety, no overlapping toxicities, and promising efficacy. Further studies are warranted to evaluate the potential role of teclistamab combination therapy on enhanced early disease control or newly diagnosed MM.

Second, another clinically relevant observation was the efficacy of teclistamab in patients previously treated with anti-BCMA therapies. Median PFS in this population was 4.5 months, which is lower than the 11.3 months observed in BCMA-naïve patients in the MajesTEC-1 RP2D cohort (10). However, our study showed that even ORRs with BCMA-targeted therapies were generally lower in patients who had prior anti-BCMA therapies as compared with BCMA-naive patients (56% vs 67%), however, the ≥VGPR and ≥CR rates showed no statistical differences. It is important to note that prior anti-BCMA-treated patients may present with more severe disease compared to BCMA-naive patients, as they are typically in a more refractory state due to the progression of the disease. As such, the outcomes of prior BCMA-treated patients were generally less favorable. For patients who achieved ≥CR after prior anti-BCMA-targeted therapy, the median duration of response (DOR) was 16.7 months, demonstrating the durability of deep responses. Additionally, in cohort C, the efficacy outcomes of patients who had previously received anti-BCMA ADC therapy were similar to those of patients who had received CAR-T therapy (ORR: 55.2% vs 53.3%) (10). A similar finding was reported in a real-world study by Dima et al. (ORR: 50% vs 57%) (39). This finding suggests that teclistamab can achieve good responses even in patients who have previously undergone T-cell redirection therapies. Furthermore, the safety profile of teclistamab in anti-BCMA-exposed patients was generally consistent with that of BCMA-naïve patients. Overall, our data suggest that teclistamab remains a viable treatment option following BCMA-targeted ADC or CAR-T therapy. BCMA loss may be a potential mechanism of primary resistance to teclistamab after BCMA-directed treatments (55). Therefore, combining teclistamab with agents such as talquetamab (a bispecific antibody targeting the novel myeloma antigen GPRC5D) may improve outcomes by overcoming resistance mechanisms, such as antigen escape, and enhancing survival in this subgroup of patients.

Furthermore, in July 2024, Johnson & Johnson announced that the marketing application for a teclistamab injection had been approved by the National Medical Products Administration (NMPA) of China, therefore, our study included the only reported Asian (China) cohort to evaluate the differences in the efficacy of teclistamab across ethnicities. Compared to the pivotal recommended phase 2 dose (RP2D) cohorts, while the baseline characteristics of the China cohort were generally consistent, some numerical differences were observed (56).The China cohort included fewer patients aged ≥75 years (7.7% vs 14.5%), fewer penta-exposed patients (53.8% vs 70.3%), and fewer patients with prior transplantation (11.5% vs 81.8%). In contrast, a higher proportion of patients in the China cohort presented with baseline features associated with a poorer prognosis, including high-risk cytogenetics (57.7% vs 25.7%), ≥1 extramedullary plasmacytoma (34.6% vs 17.0%), and ISS stage 3 disease (26.9% vs 12.3%). Despite these differences, the China cohort demonstrated a higher ORR rate (77%), and all patients achieved ≥VGPR. With a median follow-up of 15 months, the median DOR, PFS, and OS were not reached. The 12-month DOR, PFS, and OS rates were 78.5%, 68%, and 83.5%, respectively, demonstrating that Chinese patients treated with teclistamab can achieve deep and durable responses (32). Although the AE rate was higher than in the Western populations, no patients experienced a dose reduction or discontinuation due to AEs. The AEs decreased over time and were clinically managed with supportive care. Although some PIs, IMiDs, and monoclonal antibody drugs have been approved in China, unmet treatment needs still exist for patients with RRMM. Older MM patients, those with comorbidities such as renal impairment, patients with extramedullary involvement, and high-risk patients who relapse after transplantation require innovative treatments like teclistamab. However, studies in Asian populations remain limited, and more robust clinical research is needed to confirm the efficacy of teclistamab. In the future, we look forward to the publication of more data on teclistamab in Asian populations to further support its feasibility as a treatment option for RRMM.

In addition, compared with a recently published systematic review and meta-analysis by Qureshi et al., our current meta-analysis includes more studies, encompassing 4,064 patients (57). This notable difference in the number of included studies and patients, despite only a 4-month difference in search cut-off dates, can be attributed to the broader scope of our review. We systematically searched ClinicalTrials.gov and included relevant conference abstracts to capture the most recent and comprehensive evidence. Furthermore, our analysis also incorporated studies investigating teclistamab in combination regimens, providing a more extensive overview of its clinical potential. Therefore, our work not only complements the findings of Qureshi et al. but also further supports the growing body of evidence that highlights teclistamab as a promising and increasingly studied therapeutic option for patients with RRMM.

Our study had some limitations. First, the data for teclistamab in the pairwise meta-analysis mainly came from the MajesTEC-1, so there was unavoidable data redundancy. Second, due to the relatively short time since the approval of the drug, the follow-up periods in all real-world studies were relatively brief, which may have imposed certain limitations on our findings. Third, the heterogeneity in the results largely stemmed from differences in sample sizes and baseline characteristics among studies. At this stage, there is still a lack of large-scale, head-to-head randomized controlled trials to definitively establish the therapeutic advantages of teclistamab. Although this study did not fully meet all the above limitations, the overall risk of bias in study quality was considered acceptable.

## 5 Conclusion

Teclistamab has demonstrated favorable efficacy in real-world studies and clinical trials and remains a viable and effective treatment option for patients with RRMM previously exposed to BCMA-targeted therapy. Additionally, teclistamab combination therapies can improve response rates and maintain a favorable safety profile, offering new hope for overcoming BCMA resistance. Additionally, compared to Western populations, the China cohort showed better clinical benefits, although they were associated with a higher incidence of AEs. Therefore, we eagerly anticipate the future application of teclistamab in Asian RRMM populations, with the hope of bringing more treatment options and hope to patients in need. Our research indirectly supports the potential of teclistamab in clinical applications. However, there is still a lack of direct head-to-head studies to demonstrate the efficacy, therefore, we call for more direct comparative clinical trials or real-world studies in the future to validate this conclusion.

## Data Availability

The original contributions presented in the study are included in the article/**Supplementary Material**. Further inquiries can be directed to the corresponding author.
