# Supplementary material for: Effectiveness and safety of teclistamab for relapsed or refractory multiple myeloma: a systematic review and meta-analysis
Source: Front Immunol. 2025 Apr 25;16:1565407. doi: 10.3389/fimmu.2025.1565407 (PMC12061972; doi:10.3389/fimmu.2025.1565407)
Supplement: Supplementary file 1 [file DataSheet1.zip › Supplementary Material.docx]

**Supplementary Figures:**

[**Figure S1. Subgroup analysis of OS in compared studies displayed by forest plots.**	5](#_Toc188528557)

[**Figure S2. The pooled (A)ORR and (B) ≥ CR after excluding Dima, D.2024 (Baseline characteristics imbalanced).**	6](#_Toc188528558)

[**Figure S3. The pooled (A) ≥ CR, (B) PR in patients treated with Teclistamab in real-world studies.**	6](#_Toc188528559)

[**Figure S4. The pooled any grades (A) anemia, (B) infection and (C) neutropenia; The pooled risk of grade ≥ 3 (D) anemia, (E) infection, (F) neutropenia in patients treated with Teclistamab in real-world studies.**	6](#_Toc188528560)

[**Figure S5. Subgroup analysis of (A) ORR, (B) ≥ VGPR, (C) ≥ CR in Teclistamab mono therapies and combined therapies.**	8](#_Toc188528561)

[**Figure S6. Subgroup analysis of adverse effects in Teclistamab mono and multi therapy: The pooled risk of any grades (A) anemia, (B) CRS, (C) ICANS, (D) infection and (E) neutropenia; The pooled risk of grade ≥ 3 (F) anemia, (G) CRS, (H) ICANS, (I) infection and (J) neutropenia.**	8](#_Toc188528562)

[**Figure S7. Subgroup analysis of (A) ORR, (B) ≥ VGPR, (C) ≥ CR, (D) VGPR, (E) PR in clinical trials and real-world studies.**	10](#_Toc188528563)

[**Figure S8. Subgroup analysis of adverse effects in Teclistamab clinical trials and real-world studies: (A) any grades anemia, (B) any grades CRS, (C) any grades ICANS, (D) any grades infection, (E) any grades neutropenia, (F) grade ≥ 3 anemia, (G) grade ≥ 3 CRS, (H) grade ≥ 3 ICANS, (I) grade ≥ 3 infection and (J) grade ≥ 3 neutropenia.**	11](#_Toc188528564)

[**Figure S9. Subgroup analysis of (A) ORR, (B) ≥ VGPR, (C) ≥ CR，(D) any grades anemia, (E) any grades CRS, (F) any grades ICANS, (G) any grades infection, (H) any grades neutropenia in Western population and China cohort.**	14](#_Toc188528565)

[**Figure S10. Subgroup analysis of (A) ORR, (B) ≥ VGPR, (C) ≥ CR, (D) VGPR, (E) PR in anti-BCMA exposed population and non-BCMA exposed cohort.**	15](#_Toc188528566)

[**Figure S11. Subgroup analysis of adverse effects in Teclistamab anti-BCMA exposed population and non-BCMA exposed cohort: (A) any grades anemia, (B) any grades CRS, (C) any grades ICANS, (D) any grades infection, (E) any grades neutropenia, (F) grade ≥ 3 anemia, (G) grade ≥ 3 CRS, (H) grade ≥ 3 ICANS, (I) grade ≥ 3 infection and (J) grade ≥ 3 neutropenia.**	16](#_Toc188528567)

[**Figure S12. Sensitivity analysis of (A) OS, (B) PFS in compared studies displayed by forest plots.**	19](#_Toc188528578)

[**Figure S13. Funnel plots of (A) OS, (B) PFS in compared studies.**	19](#_Toc188528579)

[**Table S1. Result of a search of the selected databases (14 November 2024)**	20](#_Toc188528580)

[**Table S2. Treatment regimens and efficacy outcomes of included studies**	22](#_Toc188528581)

[**Table S3. The incidence of adverse events for patients with RRMM.**	27](#_Toc188528582)

[**Table S4. List of excluded studies, with reasons for exclusion after full text reading.**	29](#_Toc188528583)

**Figure S1. Subgroup analysis of OS in compared studies displayed by forest plots.**

A. **B.**

W: Inverse probability of treatment weighting adjusted for imbalances in prognostic baseline characteristics; NW: no weighting adjustment; Maj: Teclistamab data all comes from MajesTEC-1; RW: Teclistamab data all comes from real world studies.

**Figure S2. The pooled (A)ORR and (B) ≥ CR after excluding Dima, D.2024 (Baseline characteristics imbalanced).**

A.
 **B.**

**Figure S3. The pooled (A) ≥ CR, (B) PR in patients treated with Teclistamab in real-world studies.**

A. **B.**

**Figure S4. The pooled any grades (A) anemia, (B) infection and (C) neutropenia; The pooled risk of grade ≥ 3 (D) anemia, (E) infection, (F) neutropenia in patients treated with Teclistamab in real-world studies.**

A. **D.**

B. **E.**

C. **F.**

**Figure S5. Subgroup analysis of (A) ORR, (B) ≥ VGPR, (C) ≥ CR in Teclistamab mono therapies and combined therapies.**

**A.** **B**. **C.**

**Figure S6. Subgroup analysis of adverse effects in Teclistamab mono and multi therapy: The pooled risk of any grades (A) anemia, (B) CRS, (C) ICANS, (D) infection and (E) neutropenia; The pooled risk of grade ≥ 3 (F) anemia, (G) CRS, (H) ICANS, (I) infection and (J) neutropenia.**

A. **B. C.**

**D. E.** **F.**

**G. H. I.**

**J.**

**Figure S7. Subgroup analysis of (A) ORR, (B) ≥ VGPR, (C) ≥ CR, (D) VGPR, (E) PR in clinical trials and real-world studies.**

A. **B. C.**

**D. E.**

**Figure S8. Subgroup analysis of adverse effects in Teclistamab clinical trials and real-world studies: (A) any grades anemia, (B) any grades CRS, (C) any grades ICANS, (D) any grades infection, (E) any grades neutropenia, (F) grade ≥ 3 anemia, (G) grade ≥ 3 CRS, (H) grade ≥ 3 ICANS, (I) grade ≥ 3 infection and (J) grade ≥ 3 neutropenia.**

**A. B. C.**

**D. E. F.**

**G. H. I.**

**J.**

**Figure S9. Subgroup analysis of (A) ORR, (B) ≥ VGPR, (C) ≥ CR，(D) any grades anemia, (E) any grades CRS, (F) any grades ICANS, (G) any grades infection, (H) any grades neutropenia in Western population and China cohort.**

A. **B. C.**

D. **E. F.**

**G. H.**

**Figure S10. Subgroup analysis of (A) ORR, (B) ≥ VGPR, (C) ≥ CR, (D) VGPR, (E) PR in anti-BCMA exposed population and non-BCMA exposed cohort.**

**A. B. C.**

**D. E.**

**Figure S11. Subgroup analysis of adverse effects in Teclistamab anti-BCMA exposed population and non-BCMA exposed cohort: (A) any grades anemia, (B) any grades CRS, (C) any grades ICANS, (D) any grades infection, (E) any grades neutropenia, (F) grade ≥ 3 anemia, (G) grade ≥ 3 CRS, (H) grade ≥ 3 ICANS, (I) grade ≥ 3 infection and (J) grade ≥ 3 neutropenia.**

A. **B.**

**C. D.**

**E. F.**

**G. H.**

I. **J.**

**Figure S12. Sensitivity analysis of (A) OS, (B) PFS in compared studies displayed by forest plots.**

**Figure S13. Funnel plots of (A) OS, (B) PFS in compared studies.**

**Table S1. Result of a search of the selected databases (14 November 2024)**

| **Databases** | **Search terms** | **Results** |
| --- | --- | --- |
| PubMed | #1: "multiple myeloma"[MeSH Terms] OR ("multiple"[All Fields] AND "myeloma"[All Fields]) OR "multiple myeloma"[All Fields] OR ("multiple myeloma"[MeSH Terms] OR ("multiple"[All Fields] AND "myeloma"[All Fields]) OR "multiple myeloma"[All Fields] OR ("kahler"[All Fields] AND "disease"[All Fields]) OR "kahler disease"[All Fields]) OR ("multiple myeloma"[MeSH Terms] OR ("multiple"[All Fields] AND "myeloma"[All Fields]) OR "multiple myeloma"[All Fields] OR ("plasma"[All Fields] AND "cell"[All Fields] AND "myeloma"[All Fields]) OR "plasma cell myeloma"[All Fields]) OR ("multiple myeloma"[MeSH Terms] OR ("multiple"[All Fields] AND "myeloma"[All Fields]) OR "multiple myeloma"[All Fields] OR "myelomatoses"[All Fields]) | 67043 |
|  | #2: "Teclistamab"[All Fields] OR "JNJ-64007957"[All Fields] OR ("antibodies, bispecific"[MeSH Terms] OR ("antibodies"[All Fields] AND "bispecific"[All Fields]) OR "bispecific antibodies"[All Fields] OR ("bispecific"[All Fields] AND "antibody"[All Fields]) OR "bispecific antibody"[All Fields]) | 7909 |
|  | #3: #1 AND #2 | 581 |
| WOS | #1: ALL= (Multiple Myeloma OR Kahler Disease OR Plasma Cell Myeloma OR Myelomatose) | 86921 |
|  | #2: ALL= (Teclistamab OR JNJ-64007957 OR Bispecific antibody) | 9239 |
|  | #3: #1 AND #2 | 822 |
| Embase | #1: 'multiple myeloma' OR 'kahler disease'/exp OR 'plasma cell myeloma'/exp OR myelomatoses | 120,262 |
|  | #2: 'teclistamab'/exp OR 'jnj 64007957'/exp OR 'bispecific antibody'/exp | 7768 |
|  | #3: #1 AND #2 | 1134 |
| Cochrane Library | #1: (Multiple Myeloma OR Kahler Disease OR Plasma Cell Myeloma OR Myelomatoses) (Word variations have been searched) | 6980 |
|  | #2: (Teclistamab OR JNJ-64007957 OR Bispecific antibody) (Word variations have been searched) | 478 |
|  | #3: #1 AND #2 | 58 |
| ClinicalTrials.gov | #1: Multiple Myeloma OR Kahler Disease OR Plasma Cell Myeloma OR myelomatosis | 3610 |
|  | #2: Teclistamab OR JNJ-64007957 OR Bispecific antibody | 538 |
|  | #3: #1 AND #2 | 79 |

**Table S2. Treatment regimens and efficacy outcomes of included studies**

| **Comparative studies** | | | | | | | | | | | | | | | | | | | | | | | | | | | | |
| --- | --- | --- | --- | --- | --- | --- | --- | --- | --- | --- | --- | --- | --- | --- | --- | --- | --- | --- | --- | --- | --- | --- | --- | --- | --- | --- | --- | --- |
| **Author** | **Drug name** | | | | **Data Source** | | **Patient**  **(n)** | **HR (95% CI)** | | | | | | | | | | | **RR^a^/OR^b^ (95% CI)** | | | | | | | | | |
|  |  |  |  |  |  |  |  | **OS** | | | **PFS** | | **TTNT** | | | **DOR** | | | **ORR** | | | | **≥ CR** | | | **≥ VGPR** | | |
| Bahlis, N. J. | Tec | | | | MajesTEC-1 | | 37 | 0.52  (0.28-0.95) | | | 0.58  (0.30-1.11) | | NR | | | 0.04  (0.01-0.10) | | | 3.14^b^  (1.48-6.69) | | | | 16.3 ^b^  (3.5-77.1) | | | NR | | |
|  | Sel+Dex | | | | NCT02336815 | | 122 |  |  |  |  |  |  |  |  |  |  |  |  |  |  |  |  |  |  |  |  |  |
| Delforge, M. | Tec | | | | MajesTEC-1 | | 165 | 0.55  (0.40-0.76) | | | 0.39  (0.29-0.52) | | 0.35  (0.27-0.47) | | | NR | | | 2.31 ^a^  (1.69-3.15) | | | | NR | | | 5.97 ^a^  (3.80-9.37) | | |
|  | BelgianRWPC | | | | BELCOMM | | 112 |  |  |  |  |  |  |  |  |  |  |  |  |  |  |  |  |  |  |  |  |  |
| Krishnan, A. | Tec | | | | MajesTEC-1 | | 165 | 0.82  (0.59-1.14) | | | 0.43  (0.33-0.56) | | 0.36  (0.27-0.49) | | | NR | | | NR | | | | NR | | | NR | | |
|  | RWPC | | | | The Flatiron MM cohort | | 195 |  |  |  |  |  |  |  |  |  |  |  |  |  |  |  |  |  |  |  |  |  |
| Mateos,M.V | Tec | | | | MajesTEC-1 | | 165 | 0.54  (0.40-0.73) | | | 0.59  (0.46-0.78) | | 0.32  (0.24-0.42) | | | NR | | | 4.81 ^b^  (3.04-7.72) | | | | NR | | | 12.07 ^b^  (6.91-22.1) | | |
|  | Dara trails | | | | APOLLO, POLLUX  CASTOR, EQUULEUS | | 264 |  |  |  |  |  |  |  |  |  |  |  |  |  |  |  |  |  |  |  |  |  |
| Moreau, P. | Tec | | | | MajesTEC-1 | | 165 | 0.88  (0.55-1.41) | | | 0.63  (0.38-1.05) | | NR | | | 0.27  (0.13-0.58) | | | 1.85 ^b^  (0.88-3.89) | | | | 4.45 ^b^  (1.76-11.20) | | | 3.35 ^b^  (1.52-7.39) | | |
|  | BM | | | | DREAMM-2 | | 97 |  |  |  |  |  |  |  |  |  |  |  |  |  |  |  |  |  |  |  |  |  |
| Moreau, P. | Tec | | | | MajesTEC-1 | | 165 | 0.69  (0.50-0.95) | | | 0.49  (0.37-0.66) | | NR | | | 0.43  (0.26-0.71) | | | 2.41 ^a^  (1.80-3.23) | | | | 132.32 ^a^  (19.06-918.47) | | | 5.91^a^  (3.93-8.88) | | |
|  | RWPC | | | | LocoMMotion  MoMMent | | 302 |  |  |  |  |  |  |  |  |  |  |  |  |  |  |  |  |  |  |  |  |  |
| Rakesh Popat | Tec | | | | MajesTEC-1 | | 165 | 0.52  (0.36-0.74) | | | NR | | 0.56  (0.40-0.79) | | | NR | | | NR | | | | NR | | | NR | | |
|  | Pom+Dex | | | | NCRAS | | 645 |  |  |  |  |  |  |  |  |  |  |  |  |  |  |  |  |  |  |  |  |  |
| Dima, D. | Tec | | | | Real-word data | | 45 | Not reached  (3.9-NE) | | | 2months  (1.1-NE) | | NR | | | NR | | | 21 (47%) | | | | 9 (20%) | | | NR | | |
|  | CAR-T | | | |  |  | 65 | 12.9months  (9.5-NE) | | | 6.5months  (5.1-9.6) | |  |  |  |  |  |  | 50 (77%) | | | | 27 (42%) | | | NR | | |
| Song, J. | Tec | | | | Real-word data | | 458 | 5.08  (1.70-15.2) | | | NR | | NR | | | NR | | | NR | | | | NR | | | NR | | |
|  | CAR-T | | | |  |  | 391 |  |  |  |  |  |  |  |  |  |  |  |  |  |  |  |  |  |  |  |  |  |
| **Single-arm Clinical trials** | | | | | | | | | | | | | | | | | | | | | | | | | | | | |
| **Author** | | **Drug name** | | **Data Source** | | **Patient**  **(n)** | | | | **OS**  **(months)**  **Median**  **(95% CI)** | | **PFS**  **(months)**  **Median**  **(95% CI)** | | | **DOR**  **(months)**  **Median**  **(95% CI)** | | | **ORR** | | | **≥ CR** | | **≥ VGPR** | | **VGPR** | | | **PR** |
| Moreau, P. | | Tec | | MajesTEC-1 | | 165 | | | | 18.3  (15.1-NE) | | 11.3  (8.8-17.1) | | | 18.4  (14.9-NE) | | | 63% | | | 39.4% | | 58.8% | | 19.4% | | | 4.2% |
| Rodríguez-Otero, P. | | Tec+Dara | | NCT04108195 | | 46 | | | | NR | | NR | | | Not reached | | | 78% | | | 24.3% | | 73% | | 48.7% | | | 5% |
| Cohen, Y. C. | | Tec+Tal | | NCT04586426 | | 63 | | | | NR | | NR | | | NR | | | 84% | | | 34% | | NR | | NR | | | NR |
| Donk, N.1 | | Tec | | MajesTEC-1  Subgroup  analysis | | > 3 LOT: 122 | | | | 17.7  (12.2-NE) | | 9.7  (6.4-13.1) | | | NR | | | 58.1% | | | 41% | | NR | | NR | | | NR |
|  |  |  |  |  |  | ≤ 3 LOT: 43 | | | | 25.9  (18.3-NE) | | 18.1  (13.8-26.9) | | | NR | | | 74.4% | | | 59% | | NR | | NR | | | NR |
| Donk, N.2 | | Tec | | MajesTEC-1 update | | 165 | | | | 21.9  (16.0-NE) | | 12.5  (8.8-17.2) | | | 24  (16.2-NE) | | | NR | | | NR | | NR | | NR | | | NR |
| Searle, E. | | Tec  Dara  Len | | MajesTEC-2  NCT04722146 | | 32 | | | | NR | | NR | | | NR | | | 0.72 mg/kg:  100% | | | NR | | 0.72 mg/kg: 92.35% | | NR | | | NR |
|  |  |  |  |  |  |  |  |  |  |  |  |  |  |  |  |  |  | 1.5 mg/kg:  81.3% | | |  |  | NR | |  |  |  |  |
| Du juan | | Tec | | MajesTEC-1  China cohort | | 26 | | | | Not reached | | Not reached | | | Not reached | | | 76.9% | | | 57.7% | | 76.9% | | 19.2% | | | 0% |
| Garfall, A. L. | | Tec | | MajesTEC-1 update | | 165 | | | | 22.2 | | 11.4 | | | 24 | | | 63% | | | 46.1% | | NR | | NR | | | NR |
| J Costa, L. | | Tec | | MajesTEC-1  Subgroup  analysis | | ≥ 75 years:  24 | | | | NR | | NR | | | Not reached (9.1-NE) | | | 54.2% | | | 41.7% | | NR | | NR | | | NR |
|  |  |  |  |  |  | ISS stage III:  20 | | | | NR | | NR | | | Not reached (5.8-NE) | | | 35% | | | 15% | | NR | | NR | | | NR |
|  |  |  |  |  |  | High Cytogenic risk :38 | | | | NR | | NR | | | 18.7  (9.1-26.7) | | | 60.5% | | | 42.1% | | NR | | NR | | | NR |
|  |  |  |  |  |  | EMD:28 | | | | NR | | NR | | | Not reached (1.8-NE) | | | 35.7% | | | 17.9% | | NR | | NR | | | NR |
|  |  |  |  |  |  | BMPCs≥60%: 18 | | | | NR | | NR | | | 20.1  (5.8-NE) | | | 44.4% | | | 27.8% | | NR | | NR | | | NR |
|  |  |  |  |  |  | Penta-drug refractory: 50 | | | | NR | | NR | | | NR  (18.1-NE) | | | 60% | | | 48% | | NR | | NR | | | NR |
| Touzeau, C. | | Tec | | MajesTEC-1  Anti-BCMA exposed | | 40 | | | | 15.5  (8.3-27.9) | | 4.5  (1.3-11.6) | | | 14.8  (8-22.6) | | | 52.5% | | | 30% | | 47.5% | | 17.5% | | | 55% |
| Donk, N. | | Tec+Toci | | NCT05972135 | | 24 | | | | NR | | NR | | | NR | | | 73% | | | NR | | 59% | | NR | | | NR |
| **Real world experiences** | | | | | | | | | | | | | | | | | | | | | | | | | | | | |
| **Author** | | | **Country** | | **Patient**  **(n)** | | | | **OS**  **(months)**  **Median**  **(95% CI)** | | **PFS**  **(months)**  **Median**  **(95% CI)** | | | **DOR**  **(months)**  **Median**  **(95% CI)** | | | **ORR** | | | **≥ CR** | | **≥ VGPR** | | **VGPR** | | | **PR** | |
| Uttervall, K. | | | Sweden | | 17 | | | | NR | | Not reached | | | NR | | | 60% | | | 20% | | 53% | | 33% | | | 7% | |
| Asoori, S. | | | USA | | 37 | | | | NR | | NR | | | NR | | | 76.5% | | | NR | | NR | | NR | | | NR | |
| Dima, D. | | | USA | | > 70: 33 | | | | NR | | 5.4  (2.8-NE) | | | NR | | | 70% | | | 30% | | NR | | NR | | | NR | |
|  |  |  |  |  | ≤ 70: 69 | | | | NR | | 3.6  (2.0-NE) | | | NR | | | 61% | | | 28% | | NR | | NR | | | NR | |
| Gordon, B. | | | USA | | 45 | | | | NR | | NR | | | NR | | | 48.9% | | | NR | | 22.2% | | NR | | | 26.7% | |
| Grajales-Cruz, A. F. | | | USA | | 22 | | | | NR | | NR | | | NR | | | 63% | | | 36% | | NR | | NR | | | NR | |
| Maringanti, S. A. | | | USA, Greece, Spain | | All: 61 | | | | NR | | NR | | | NR | | | 60% | | | 11.9% | | 45.8% | | 33.9% | | | 13.6% | |
|  |  |  |  |  | Anti-BCMA exposed: 31 | | | |  |  |  |  |  |  |  |  | 61% | | | NR | | 48.1% | | 45.2% | | | 12.9% | |
| Dima, D. | | | USA | | All: 106 | | | | Not reached (NE-NE) | | 5.4  (3.4-NE) | | | NR | | | 66% | | | 29% | | NR | | NR | | | NR | |
|  |  |  |  |  | Anti-BCMA exposed: 56 | | | |  |  |  |  |  |  |  |  | 59% | | | 26.8% | | 45% | | 17.9% | | | 14% | |
| Firestone, R. S. | | | USA | | All: 47 | | | | NR | | NE | | | NR | | | 64% | | | NR | | 38% | | NR | | | 26% | |
|  |  |  |  |  | Anti-BCMA exposed: 26 | | | | NR | | 3.4 | | | NR | | | 50% | | | NR | | NR | | NR | | | NR | |
| Ghamsari, F. | | | USA | | 18 | | | | NE  (7.8-NE） | | 12.5  (1.0-NE)) | | | Not reached | | | 50% | | | NR | | 50% | | NR | | | 0% | |
| Mohan, M. | | | USA | | 110 | | | | Not reached | | Not reached | | | NR | | | 62% | | | 20% | | 51% | | 31% | | | 11% | |
| Riedhammer, C. | | | Germany | | All: 123 | | | | Not reached | | 8.7 | | | Not reached | | | 59.3% | | | 22% | | 48% | | 26% | | | 11.4% | |
|  |  |  |  |  | Anti-BCMA exposed: 45 | | | |  |  |  |  |  |  |  |  | 54.8% | | | NR | | NR | | NR | | | NR | |
| Tan, C. R. C. | | | USA | | 69 | | | | NR | | NR | | | Not reached | | | 62% | | | NR | | 42% | | NR | | | 20% | |

Sel, selinexor; Dex, dexamethasone; Tec, teclistamab; RWPC, real-world physician's choice; LOT, lines of therapy; Dara, daratumumab; Pom, pomalidomide; BM, Belantamab Mafodotin; CAR-T, Chimeric Antigen Receptor T-Cell; LEN, lenalidomide; Tal, talquetamab; Toci, tocilizumab; NR, not reported; NE, not estimated; BCMA, B-cell maturation antigen; ORR, overall response rate; CR, complete response; VGPR, very good partial response; PR, partial response; OS, overall survival; PFS, progression-free survival; TTNT, time to next treatment; DOR, duration of response; EMD, extramedullary disease; BMPC, bone marrow plasma cells.

**Table S3. The incidence of adverse events for patients with RRMM.**

| **Comparative studies** | | | | | | | | | | | | | | |
| --- | --- | --- | --- | --- | --- | --- | --- | --- | --- | --- | --- | --- | --- | --- |
| **Author** | **Year** | **Drug**  **name** | **Any Grade** | | | | | | **Grade ≥ 3** | | | | | |
|  |  |  | **Any AE** | **Infection** | **Neutropenia** | **Anemia** | **CRS** | **ICANS** | **Any AE** | **Infection** | **Neutropenia** | **Anemia** | **CRS** | **ICANS** |
| Dima, D. | 2024 | Tec | NR | NR | NR | NR | 60% | 15.5% | NR | NR | NR | NR | 0% | 4.5% |
|  |  | CAR-T |  |  |  |  | 72% | 27.5% |  |  |  |  | 4.5% | 4.5% |
| Song, J. | 2024 | Tec | NR | NR | NR | NR | 27.4% | 13.7% | NR | NR | NR | NR | NR | NR |
|  |  | CAR-T |  |  |  |  | 37.8% | 14.8% |  |  |  |  |  |  |
| **Single-arm Clinical trials** | | | | | | | | | | | | | | |
| **Author** | **Year** | **Drug**  **name** | **Any Grade** | | | | | | **Grade ≥ 3** | | | | | |
|  |  |  | **Any AE** | **Infection** | **Neutropenia** | **Anemia** | **CRS** | **ICANS** | **Any AE** | **Infection** | **Neutropenia** | **Anemia** | **CRS** | **ICANS** |
| Moreau, P. | 2022 | Tec | 100% | 76.4% | 70.9% | 52.1% | 72.1% | 3% | 94.5% | 44.8% | 64.2% | 37% | 0.6% | 0% |
| Rodríguez-Otero, P. | 2022 | Tec+Dara | 91% | 63% | 54% | 46% | 61% | 2% | 78% | 28% | 50% | 28% | 0% | 0% |
| Cohen, Y. C. | 2023 | Tec+Tal | NR | NR | 76% | 60% | 81% | 2% | NR | NR | 75% | 43% | 3% | 0% |
| Donk, N.2 | 2023 | Tec | NR | 78% | 72% | 54% | 72% | 3% | NR | 52% | 65% | 38% | 0.6% | 0% |
| Searle, E. | 2023 | Tec+Dara+Len | NR | 75% | 75% | NR | NR | NR | NR | 28.1% | 68.8% | NR | NR | NR |
| Du juan | 2024 | Tec | NR | 96.2% | 96.2% | 88.5% | 96.2% | 0% | NR | 69.2% | 76.9% | 53.8% | 0% | 0% |
| Garfall, A. L. | 2024 | Tec | NR | 79% | 72% | 55% | NR | NR | NR | 55% | 65% | 38% | NR | NR |
| Touzeau, C. | 2024 | Tec | 100% | 70% | 70% | 50% | 65% | 10% | NR | 42.5% | 65% | 35% | 0% | 2.5% |
| Donk, N. | 2024 | Tec+Toci | NR | 79% | 63% | 58% | 25% | 4% | NR | 25% | 63% | 25% | 0% | 0% |
| **Real world experiences** | | | | | | | | | | | | | | |
| **Author** | **Year** | **Drug**  **name** | **Any Grade** | | | | | | **Grade≥3** | | | | | |
|  |  |  | **Any AE** | **Infection** | **Neutropenia** | **Anemia** | **CRS** | **ICANS** | **Any AE** | **Infection** | **Neutropenia** | **Anemia** | **CRS** | **ICANS** |
| Uttervall, K. | 2022 | Tec | NR | 35.3% | NR | NR | 53% | NR | NR | NR | NR | NR | 0% | NR |
| Asoori, S. | 2023 | Tec | NR | 72.97% | NR | NR | NR | NR | NR | NR | 43.24% | NR | NR | NR |
| Dima, D. | 2023 | Tec > 70 | NR | 33% | NR | NR | 67% | 21% | NR | NR | 24% | 21% | 3% | 0% |
|  |  | Tec ≤ 70 | NR | 26% | NR | NR | 64% | 11% | NR | NR | 22% | 16% | 0% | 4% |
| Grajales-Cruz, A. F. | 2023 | Tec | 100% | 55% | NR | NR | 73% | 5% | NR | 50% | NR | NR | 0% | 5% |
| Maringanti, S. A. | 2023 | Tec | NR | 37% | NR | 49% | 7% | NR | NR | NR | NR | NR | 1.3% | 1.3 |
| Dima, D. | 2024 | Tec | NR | 31% | 44.4% | 66.4% | 64% | 14% | NR | 20% | 19.8% | 15% | 1% | 3% |
| Firestone, R. S. | 2024 | Tec | NR | NR | NR | NR | 52% | NR | NR | NR | NR | NR | NR | NR |
| Ghamsari, F. | 2024 | Tec | NR | 67% | NR | NR | 67% | 11% | NR | NR | NR | NR | 0% | 0% |
| Graf, K. C. | 2024 | Tec | NR | NR | NR | NR | 52% | 4% | NR | NR | NR | NR | 0% | NR |
| Kawasaki, Y. | 2024 | Overall | NR | 41% | 30% | NR | 48.1% | 14.8% | NR | NR | NR | NR | NR | NR |
|  |  | 1, 3, 5days: 23 | NR | 43% | 22% | 0% | 48% | 17% |  |  |  |  |  |  |
|  |  | 1, 4, 7days: 4 | NR | 25% | 100% | 0% | 50% | 0% |  |  |  |  |  |  |
| Mohan, M. | 2024 | Tec | NR | 40% | NR | NR | 56% | 11% | NR | 26% | NR | NR | 4.7% | 4.5% |
| Riedhammer, C. | 2024 | Tec | NR | 54.5% | 55.3% | 80.5% | 58.5% | 7.3% | NR | 26.8% | 38% | 31.7% | 1.6% | 0.8% |

Sel, selinexor; Dex, dexamethasone; Tec, teclistamab; RWPC, real-world physician's choice; Dara, daratumumab; Pom, pomalidomide; BM, Belantamab Mafodotin; CAR-T, Chimeric Antigen Receptor T-Cell; LEN, lenalidomide; Tal, talquetamab; Toci, tocilizumab; NR, not reported; AE, adverse event; CRS, cytokine release syndrome; ICANS, immune effector cell-associated neurotoxicity syndrome.

**Table S4. List of excluded studies, with reasons for exclusion after full text reading.**

| **First Author/Sponsers** | **Year** | **Title** | **Article type** | **Reason for exclusion** |
| --- | --- | --- | --- | --- |
| Janssen-Cilag | 2022 | A Clinical Study to Compare Teclistamab Monotherapy versus Pomalidomide, Bortezomib, Dexamethasone (PVd) or Carfilzomib, Dexamethasone (Kd) in Participants with Relapsed or Refractory Multiple Myeloma who have Received 1 to 3 Prior Lines of Therapy, Including an Anti-CD38 Monoclonal Antibody and Lenalidomide | Clinical trial | No results posted |
| Janssen-Cilag | 2022 | A randomized phase 3 study of teclistamab in combination with daratumumab and lenalidomide; talquetamab in combination with daratumumab and lenalidomide; versus a combination of daratumumab, lenalidomide and dexamethasone in new multiple myeloma patients who cannot undergo stem cell transplant as the first therapy | Clinical trial | Not Relapsed or Refractory Myeloma |
| Janssen-Cilag | 2022 | A Phase 3 Randomized Study Comparing Teclistamab in Combination with Daratumumab SC and Lenalidomide (Tec-DR) and Talquetamab in Combination with Daratumumab SC and Lenalidomide (Tal-DR) versus Daratumumab SC, Lenalidomide, and Dexamethasone (DRd) in Participants with Newly Diagnosed Multiple Myeloma Who are Either Ineligible or not Intended for Autologous Stem Cell Transplant as Initial Therapy | Clinical trial | Not Relapsed or Refractory Myeloma |
| Martin, T. G. | 2022 | Health-related quality of life in patients with relapsed/refractory multiple myeloma (RRMM) treated with teclistamab, a B-cell maturation antigen (BCMA) x CD3 bispecific antibody: Patient-reported outcomes in MajesTEC-1 | Conference Abstract | Not related to effectiveness or safety |
| Popat, R. | 2022 | Health-related quality of life with Teclistamab, a b-cell maturation antigen x cd3 bispecific antibody, in patients with relapsed/refractory multiple myeloma from MajesTEC-1 | Conference Abstract | Not related to effectiveness or safety |
| Sabrin Tahri | 2022 | Phase 3 Study of Teclistamab in Combination with Lenalidomide and Teclistamab Alone Versus Lenalidomide Alone in Participants with Newly Diagnosed Multiple Myeloma as Maintenance Therapy Following Autologous Stem Cell Transplantation | Clinical trial | Not Relapsed or Refractory Myeloma |
| Zamagni, E. | 2022 | MajesTEC-4 (EMN30): A Phase 3 Trial of Teclistamab plus Lenalidomide Versus Lenalidomide Alone as Maintenance Therapy Following Autologous Stem Cell Transplantation in Patients with Newly Diagnosed Multiple Myeloma | Meeting Abstract | Not Relapsed or Refractory Myeloma |
| Amandine Hugue | 2023 | Analysis of Effectiveness and Safety of Teclistamab in Relapsed and Refractory Multiple Myeloma Patients: A Collaborative Janssen - IFM Study IFM 2023-01: TEC-CARE | Clinical trial | No results posted |
| Banerjee, R. | 2023 | Evolving Real-World Characteristics and Step-up Dosing Among Early Initiators of Teclistamab for Multiple Myeloma - a National All-Payer Claims Database Study | Conference Abstract | Not related to effectiveness or safety |
| Bansal, R. | 2023 | Outpatient Management of CAR-T and Teclistamab for Patients with Lymphoma and Multiple Myeloma | Conference Abstract | Not related to effectiveness or safety |
| Janssen-Cilag | 2023 | A Phase 1 Randomized, Open Label Pharmacokinetic Comparability Study Comparing Pre- and Post-change Teclistamab in Participants with Relapsed/Refractory Multiple Myeloma | Clinical trial | No results posted |
| Janssen-Cilag | 2023 | A study comparing talquetamab plus pomalidomide, talquetamab plus teclistamab, and elotuzumab, pomalidomide, and dexamethasone or pomalidomide, bortezomib, and dexamethasone in participants with relapsed or refractory myeloma who have received an Anti-CD38 antibody and lenalidomide | Clinical trial | No results posted |
| Janssen-Cilag l | 2023 | A Phase 3 Randomized Study Comparing Talquetamab in Combination with Pomalidomide (Tal-P), Talquetamab in Combination with Teclistamab (Tal-Tec), and Investigator’s Choice of Either Elotuzumab, Pomalidomide, and Dexamethasone (EPd) or Pomalidomide, Bortezomib, and Dexamethasone (PVd) in Participants with Relapsed or Refractory Myeloma who Have Received 1 to 4 Prior Lines of Therapy Including an Anti-CD38 Antibody and Lenalidomide | Clinical trial | No results posted |
| Jeremie ZERBIT | 2023 | A Real-World Study of Bispecific Antibodies, Teclistamab and Elranatamab, for Patients with Multiple Myeloma | Clinical trial | No results posted |
| Joiner, L. | 2023 | Teclistamab in patients with multiple myeloma and impaired renal function | Letter | Not Relapsed or Refractory Myeloma |
| Lachenal, F. | 2023 | Teclistamab in Relapsed Refractory Multiple Myeloma Patients on Dialysis: A French Experience | Conference Abstract | Not related to effectiveness or safety |
| Martin, T. G. | 2023 | Detailed overview of incidence and management of cytokine release syndrome observed with teclistamab in the MajesTEC-1 study of patients with relapsed/refractory multiple myeloma | Article | Not related to effectiveness or safety |
| Miao, X. | 2023 | Population Pharmacokinetics and Exposure-Response with Teclistamab in Patients with Relapsed/Refractory Multiple Myeloma: Results from MajesTEC-1 | Article | Not related to effectiveness or safety |
| Michelle Armogan | 2023 | Teclistamab or Talquetamab in Combination with Daratumumab SC for High-Risk Smoldering Myeloma: A Clinical and Correlative Phase 2 Sequential Cohort Immuno-Oncology Study to REVIVE Early Myeloma | Clinical trial | Not Relapsed or Refractory Myeloma |
| Mohammad Nader, S. | 2023 | Characterization of Anthropometric Changes in Patients with Relapsed/Refractory Myeloma Treated with BCMA Bispecific Antibody Teclistamab | Conference Abstract | Not related to effectiveness or safety |
| Sanish Davis | 2023 | A study of Teclistamab Monotherapy versus Pomalidomide, Bortezomib, Dexamethasone or Carfilzomib, Dexamethasone in Participants with Relapsed or Refractory Multiple Myeloma | Clinical trial | No results posted |
| Snyder, J. | 2023 | Outcomes of Patients with Relapsed/Refractory Multiple Myeloma Who Received at Least 2 BCMA-Directed Therapies | Article | Not Teclistamab |
| Stacey Pundock | 2023 | Phase 2, Single-Arm, Non-Inferiority Study of Limited-Duration Teclistamab for Relapsed Refractory Multiple Myeloma | Clinical trial | No results posted |
| Usmani, S. Z. | 2023 | Durability of Responses with Biweekly Dosing of Teclistamab in Patients with Relapsed/Refractory Multiple Myeloma Achieving a Clinical Response in the MajesTEC-1 Study | Conference Abstract | Not related to effectiveness or safety |
| van de Wyngaert, Z. | 2023 | BCMA-targeting agents for refractory multiple myeloma | Review | Not Teclistamab |
| Vishwamitra, D. | 2023 | Longitudinal Correlative Profiles of Responders, Nonresponders, and Those with Relapse on Treatment with Teclistamab in the Phase 1/2 MajesTEC-1 Study of Patients with Relapsed/Refractory Multiple Myeloma | Conference Abstract | Not related to effectiveness or safety |
| Rifkin, R. M. | 2023 | Optec: A Phase 2 Study to Evaluate Outpatient Administration of Teclistamab, a BCMA-Targeting Bispecific Antibody, in Patients with Multiple Myeloma | Clinical trial | Limited data |
| Touzeau, C. | 2023 | MajesTEC-9: A randomized phase 3 study of teclistamab versus pomalidomide, bortezomib, and dexamethasone or carfilzomib and dexamethasone in patients with relapsed/refractory multiple myeloma | Clinical trial | Limited data |
| Hebraud, B. | 2023 | French Monocentric Experience of Outpatient Step-up Dosing of Teclistamab in Relapsed Refractory Multiple Myeloma | Conference Abstract | Limited data |
| Midha, S. | 2023 | Real world experience of patients treated with teclistamab: a BCMA-directed bispecific T-cell engaging therapy for multiple myeloma | Conference Abstrac | Limited data |
| Schaefers, C. | 2023 | BCMA x CD3 bispecific antibody treatment with teclistamab in relapsed and/or refractory multiple myeloma: a real-world monocentric analysis | Conference Abstrac | Limited data |
| Bansal, R. | 2024 | Outpatient Management of CAR-T and Teclistamab for Patients with Lymphoma and Multiple Myeloma | Conference Abstract | Not related to effectiveness or safety |
| Bansal, R. | 2024 | Real world outcome of patients with multiple myeloma who received bispecific antibodies after CAR-T therapy | Conference Abstract | Not Teclistamab |
| Cheng, L. | 2024 | Efficacy and safety of bispecific antibodies vs. immune checkpoint blockade combination therapy in cancer: a real-world comparison | Review | Not Relapsed or Refractory Myeloma |
| Christine Chen | 2024 | Outpatient-based Teclistamab Step-up Dosing in Patients with Relapsed/Refractory Multiple Myeloma: Process Development in Academic and Community Centres, and Evaluating Impact on Caregiver Burden | Clinical trial | Not related to effectiveness or safety |
| Janssen Research & Development, LLC | 2024 | A Phase 3 Randomized Study Comparing Talquetamab in Combination with Pomalidomide (Tal-P), Talquetamab in Combination With Teclistamab (Tal-Tec), and Investigator's Choice of Either Elotuzumab, Pomalidomide, and Dexamethasone (EPd) or Pomalidomide, Bortezomib, and Dexamethasone (PVd) in Participants With Relapsed or Refractory Myeloma Who Have Received 1 to 4 Prior Lines of Therapy Including an Anti-CD38 Antibody and Lenalidomide | Clinical trial | No results posted |
| Janssen Research & Development, LLC | 2024 | A Phase 2, Open-label Study to Evaluate the Efficacy and Safety of Different Sequences of Ciltacabtagene Autoleucel (Cilta-cel), Talquetamab SC in Combination with Daratumumab SC (Tal-D) and Teclistamab SC in Combination with Daratumumab SC (Tec-D) Following Induction With Daratumumab, Bortezomib, Lenalidomide and Dexamethasone (DVRd) in Participants With Standard-risk Newly Diagnosed Multiple Myeloma | Clinical trial | Not Relapsed or Refractory Myeloma |
| Janssen Research & Development, LLC | 2024 | A Study Comparing Talquetamab Plus Pomalidomide, Talquetamab Plus Teclistamab, and Elotuzumab, Pomalidomide, and Dexamethasone or Pomalidomide, Bortezomib, and Dexamethasone in Participants with Relapsed or Refractory Myeloma Who Have Received an Anti-CD38 Antibody and Lenalidomide | Clinical trial | No results posted |
| Lacramioara    Arvata | 2024 | A Phase I randomized, open-label pharmacokinetic comparability study comparing pre- and post-change teclistamab in participants with relapsed/refractory multiple myeloma | Clinical trial | Not related to effectiveness or safety |
| Martin, T. G. | 2024 | Teclistamab Improves Patient-Reported Symptoms and Health-Related Quality of Life in Relapsed or Refractory Multiple Myeloma: Results from the Phase II MajesTEC-1 Study | Article | Not related to effectiveness or safety |
| Moore, D. C. | 2024 | Elranatamab vs. teclistamab: battle of the BCMA bispecifics in relapsed/refractory multiple myeloma | Article | Wrong comparison |
| Nath, K. | 2024 | Comparison of infectious complications with BCMA-directed therapies in multiple myeloma | Article | Not related to effectiveness or safety |
| National Cancer Institute (NCI) | 2024 | A Phase 1b Trial of Teclistamab in Combination with Iberdomide for Relapsed/Refractory Multiple Myeloma | Clinical trial | No results posted |
| Nooka, A. K. | 2024 | Incidence, timing, and management of infections in patients receiving teclistamab for the treatment of relapsed/refractory multiple myeloma in the MajesTEC-1 study | Article | Not related to effectiveness or safty |
| Prestes-Carneiro, L. E. | 2024 | Infectious Diseases and Secondary Antibody Deficiency in Patients from a Mesoregion of São Paulo State, Brazil | Article | Not Relapsed or Refractory Myeloma |
| Reyes, K. R. | 2024 | Salvage therapies including retreatment with BCMA-directed approaches after BCMA CAR-T relapses for multiple myeloma | Article | Not related to effectiveness or safety |
| Salah, I. B. | 2024 | Impressive extramedullary plasmacytoma response in refractory multiple myeloma treated with teclistamab | Article | Not related to effectiveness or safety |
| Shrestha, A. | 2024 | Exploring T cell subsets as predictors of response to BCMA targeting bispecific antibody therapy in multiple myeloma | Conference Abstract | Not Teclistamab |
| Snyder, J. | 2024 | Patient Outcomes Following First and Second Exposure to BCMA-Directed Therapies Including CAR-T Cell Therapy in Relapsed/Refractory Multiple Myeloma | Conference Abstract | Not Teclistamab |
| Tabbara, N. | 2024 | Ambulatory teclistamab administration in patients with relapsed/refractory multiple myeloma | Article | Not related to effectiveness or safety |
| University of Washington | 2024 | Phase 1b Study of ST-067 (Decoy-Resistant IL-18) With Teclistamab in Multiple Myeloma | Clinical trial | No results posted |
| Uttervall, K. | 2024 | Effectiveness and infectious complications of BCMA T-cell engagers in treating multiple myeloma: Real-world evidence from Sweden | Article | Not Teclistamab |
| van de Donk, N. W. | 2024 | MM-344 Longer-Term Follow-up of Patients Receiving Prophylactic Tocilizumab for the Reduction of Cytokine Release Syndrome (CRS) in the Phase 1/2 MajesTEC-1 Study of Teclistamab in Relapsed/Refractory Multiple Myeloma (RRMM) | Article | Not Teclistamab |
| van de Donk, N. W. | 2024 | Impact of covid-19 on outcomes with Teclistamab in the phase 1/2 MajesTEC-1 study in patients with relapsed/refractory multiple myeloma | Article | Not related to effectiveness or safety |
| van de Donk, N. W. | 2024 | Longer-term follow-up of patients (pts) receiving prophylactic tocilizumab (toci) for the reduction of cytokine release syndrome (CRS) in the phase 1/2 MajesTEC-1 study of teclistamab in relapsed/refractory multiple myeloma (RRMM) | Article | Not Teclistamab |
| Jagannath, S. | 2024 | Indirect comparison of linvoseltamab versus teclistamab for triple-class exposed (TCE) relapsed/refractory multiple myeloma (RRMM) | Conference Abstract | Wrong comparison |
| Mol, I. | 2024 | A matching-adjusted indirect comparison of the efficacy of elranatamab versus teclistamab in patients with triple-class exposed/refractory multiple myeloma | Article | Wrong comparison |
